# Significant duration prediction of seismic ground motions using machine learning algorithms

**DOI:** 10.1371/journal.pone.0299639

**Published:** 2024-02-28

**Authors:** Xinle Li, Pei Gao

**Affiliations:** College of Civil Engineering, Dalian Minzu University, Dalian, 116600, Liaoning, China; CINVESTAV IPN: Centro de Investigacion y de Estudios Avanzados del Instituto Politecnico Nacional, MEXICO

## Abstract

This study aims to predict the significant duration (*D*_5-75_, *D*_5-95_) of seismic motion by employing machine learning algorithms. Based on three parameters (moment magnitude, fault distance, and average shear wave velocity), two additional parameters(fault top depth and epicenter mechanism parameters) were introduced in this study. The XGBoost algorithm is utilized for characteristic parameter optimization analysis to obtain the optimal combination of four parameters. We compare the prediction results of four machine learning algorithms (random forest, XGBoost, BP neural network, and SVM) and develop a new method of significant duration prediction by constructing two fusion models (stacking and weighted averaging). The fusion model demonstrates an improvement in prediction accuracy and generalization ability of the significant duration when compared to single algorithm models based on evaluation indicators and residual values. The accuracy and rationality of the fusion model are validated through comparison with existing research.

## Introduction

Duration is a crucial component of earthquake ground motion, with a significant influence on structural damage. Various definitions of earthquake duration exist, such as bracket duration, uniform duration, and significant duration. The concept of significant duration accounts for the effect of seismic energy accumulation on structures and has been extensively studied. Researchers have defined significant duration in multiple ways. Husid [1] identified the point at which the Husid curve stabilizes as the end of the strong motion segment. Donovan [2] selected the time interval during which Arias intensity accumulates from 0 to 90% as the significant duration. Trifunac [3] discovered that the energy growth rate of the initial and final segments of the Husid curve is slow, and defined the time interval during which Arias intensity accumulates from 5% to 95% as the significant duration (*D*_5-95_). Somerville [4] proposed using 5%-75% as the threshold for significant duration (*D*_5-75_). *D*_5-95_ and *D*_5-75_ are currently the most widely accepted definitions of significant duration.

Existing research predominantly predicts duration by establishing regression equations based on earthquake data classification. Kempton [5] considered the influence of local site conditions on duration, gathered seismic records for regression analysis, and derived a fitting equation for significant duration. Building upon Kempton’s work, Stafford [6] considered influential parameters (moment magnitude *M*_w_, rupture distance *R*_rup_, average shear wave velocity of soil layer *V*_s30_, depth to the top of the fault *Z*_tor_) and provided fitting equations applicable to *D*_5-95_ and *D*_5-75_ significant durations. Lee [7] obtained fitting equations for significant duration by comparing earthquake ground motion data from two different regions, examining the differences between them. Boore [8] proposed a path duration fitting equation based on NGA-West2 data, applicable to significant duration. Afshari [9] considered the seismic epicenter mechanism and derived a fitting formula for significant duration applicable to seismically active crustal regions. Xu [10] proposed a fitting equation for significant duration applicable to China based on Bommer’s research.

As modern earthquake observation technologies advance rapidly, traditional methods for studying earthquake ground motion parameters prove inadequate in efficiently analyzing large data volumes and minimizing the influence of human classification. However, the swift progress of artificial intelligence technology has facilitated addressing these challenges. Machine learning algorithms are increasingly employed in the study of earthquake ground motion parameters, owing to their rapid processing, high accuracy, and effectiveness in solving nonlinear coupling problems.Yu [11] utilized the support vector machine algorithm to create a peak ground motion prediction model based on the Japanese KiK-net database, discovering that the overall error standard deviation of the SVM-based prediction model for peak ground motion in the training and testing sets was smaller than that of conventional *P*_d_-based prediction models. Arjun [12] applied the neural network algorithm to establish a prediction model for the *D*_5-95_ significant duration based on the Japanese KiK-net database, achieved satisfactory results. Based on the data of KiK-net database in Japan, Hammal [13] adopted neural network algorithm and introduced the concept of bearing angle. The results showed that the predicted value of significant duration based on neural network has good correlation with the observed value. Sensitivity analysis showed that moment magnitude and source depth are the main factors affecting significant duration, while epicentral distance and shear wave velocity have little influence on significant duration. Chanda [14] developed prediction models for significant duration using neural networks, decision trees, random forests, Adaboost, and SVM based on the earthquake ground motion database in Chile, discovering that the tree-based model exhibited the best prediction performance.

### Earthquake ground motion data

For this study, we selected 3592 earthquake records from 1983 stations, based on the NGA-West2 strong-motion database, with a magnitude of 4.0 or higher and a fault distance of less than 100km.It is important to note that the earthquake ground motion data used in this study is specific to shallow crustal earthquakes. Each record contains two horizontal components and one vertical component. Site conditions were classified based on the average shear wave velocity (*V*_s30_) as specified by the US NEHRP. The number of seismic recording groups in each site was 54, 1979, 1506 and 53 groups in Class B, C, D and E, respectively.

**Fig 1:**
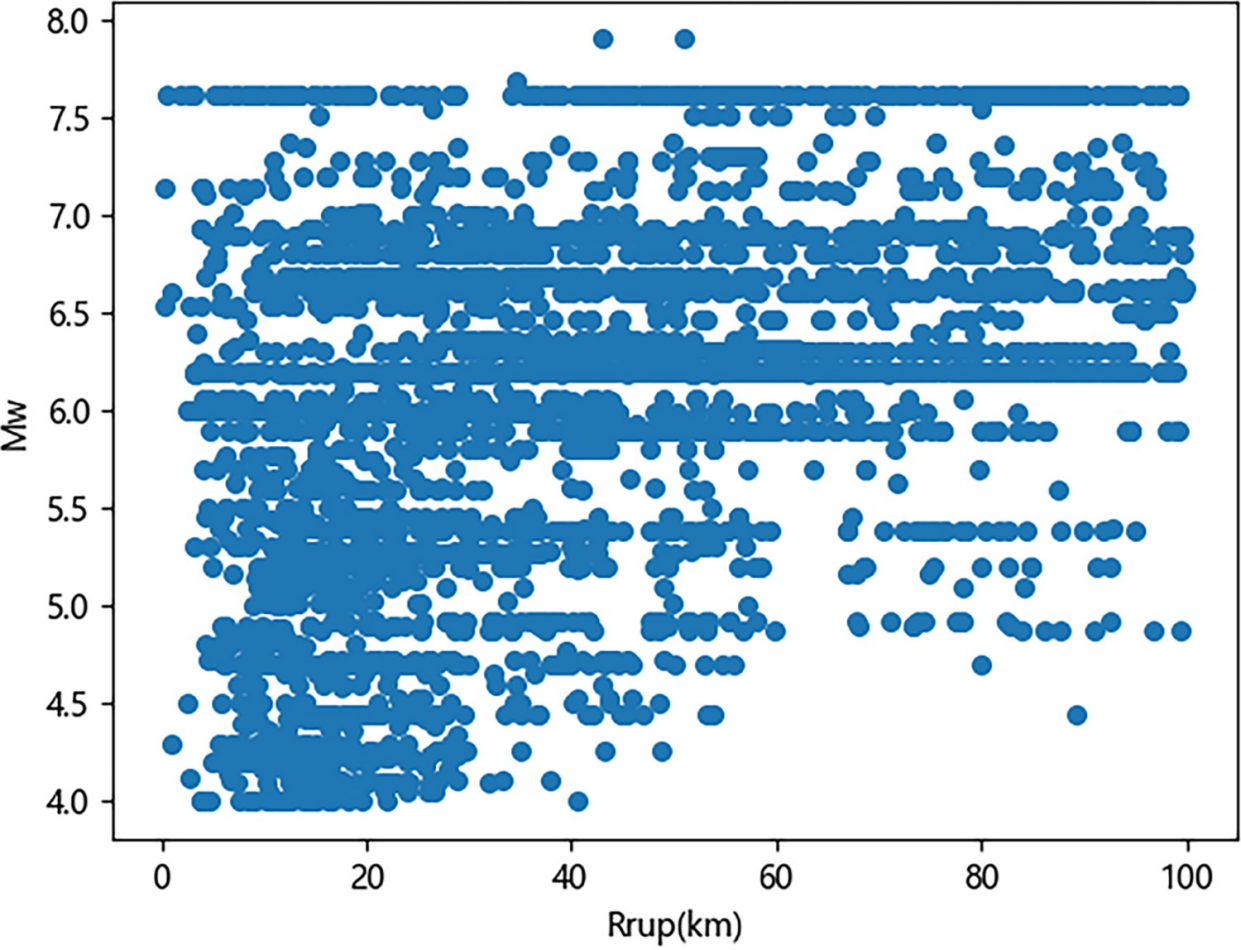
Significant duration distribution with *M*_w_ and *R*_rup_.

**Fig 2:**
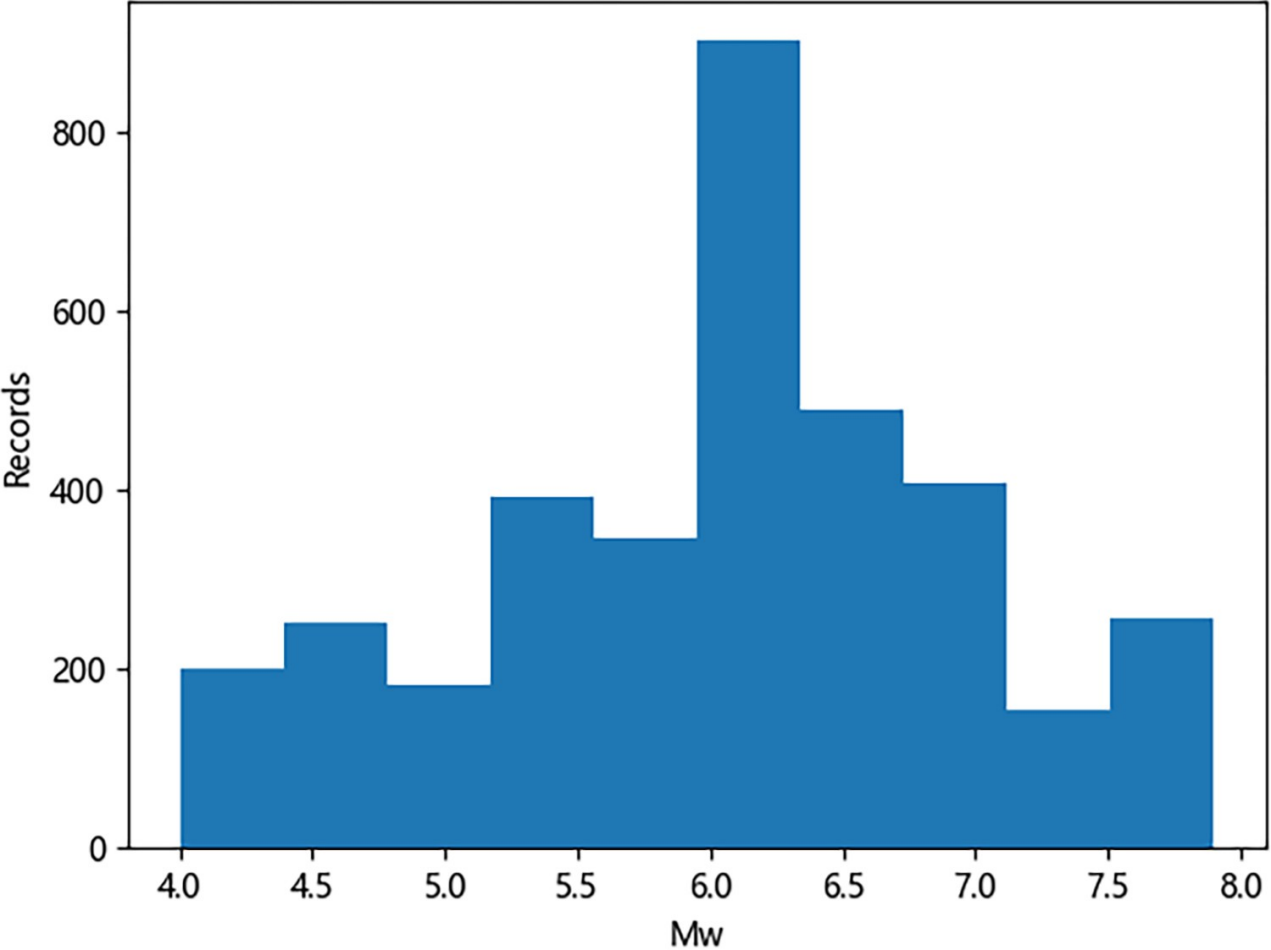
Significant duration distribution with *M*_w_.

**Fig 3:**
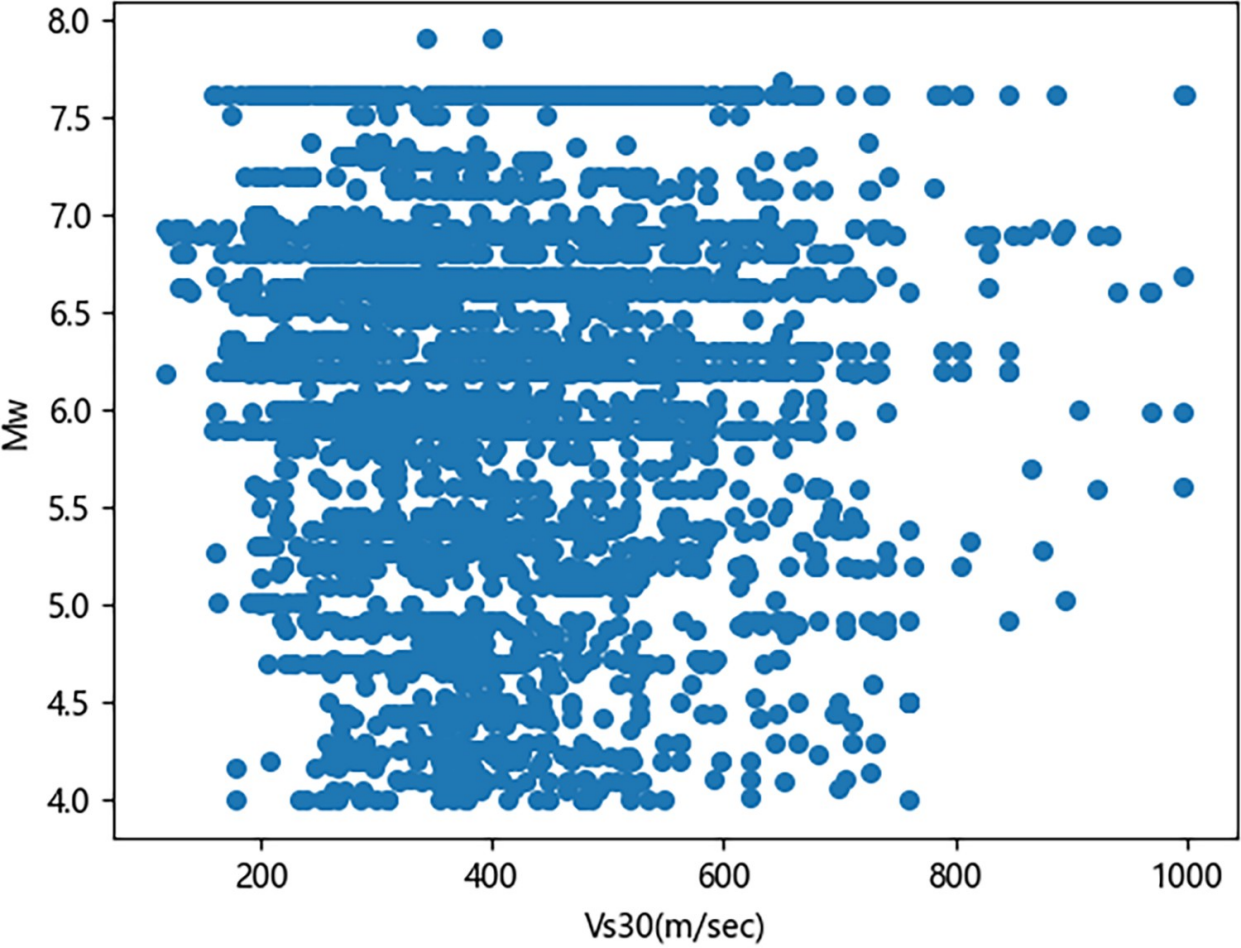
Significant duration distribution with *M*_w_ and *V*_*s30*_.

**Fig 4:**
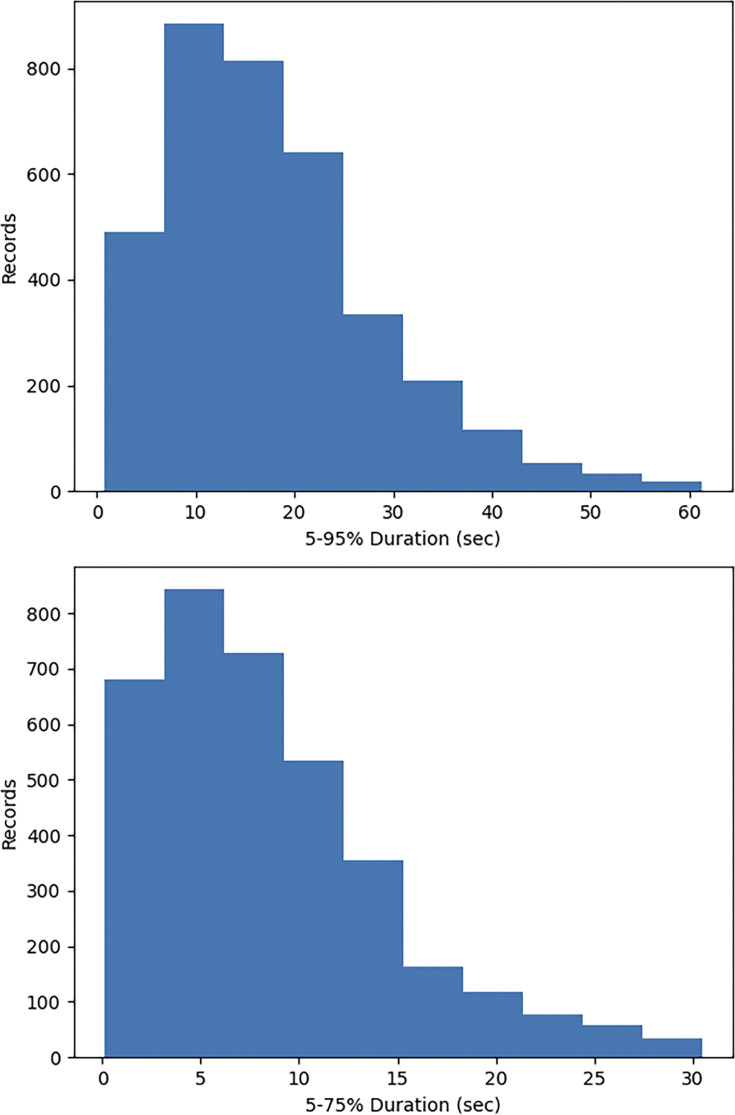
Significant duration histograms for *D*_5-95_ and *D*_5-75_. _(a)_ *D*_5-95_ histogram (b) *D*_5-75_ histogram.

Mention that while velocity time histories have been considered in previous studies, this work focuses on acceleration time histories. Figs 1–3 illustrate the distribution of earthquake records concerning magnitude *M*_w_, fault distance *R*_*rup*_, and *V*_s30_. As observed in the figures, a relatively high proportion of medium to large earthquake records with magnitudes ranging from 5.2 to 7.0, while the number of records for magnitudes between 4–5.2 and 7–8 is comparatively small. The average shear wave velocity of the sites is primarily concentrated within the range of 100-800m/s, with Classes C and D being predominant, and the data distribution is relatively uniform across all magnitudes and fault distances. Fig 4 shows two kinds of significant duration (*D*_5-95_, *D*_5-75_) histograms obtained from the acceleration time history, and it can be seen that the duration distribution basically assumes normal distribution.

### Establishment of machine learning models

#### Evaluation index

In this study, three evaluation index, namely, variance (*R*^2^), mean squared error (MSE), and mean absolute error (MAE), are utilized to assess the effectiveness of the models or regression equations.


$$R^{2}=1-\frac{\sum_{i=1}^{n}{\left({\overset{⌢}{y}}_{i}-{\bar{y}}_{i}\right)}^{2}}{\sum_{i=1}^{n}{\left(y_{i}-{\overset{⌢}{y}}_{i}\right)}^{2}}$$ (1)

$$\mathrm{MSE}=\frac{1}{n}\sum_{i=1}^{n}{\left(y_{i}-{\overset{⌢}{y}}_{i}\right)}^{2}$$ (2)

$$\mathrm{MAE}=\frac{1}{n}\sum_{i=1}^{n}\left|\left(y_{i}-{\overset{⌢}{y}}_{i}\right)\right|$$ (3)

Where: *n* represents the sample size, *y*_*i*_ represents the actual observed value, ${\overset{⌢}{y}}_{i}$ represents the predicted value, and ${\bar{y}}_{i}$ represents the mean of the actual observed values.

The *R*^2^ value ranges from 0 to 1, with a value closer to 1 indicating better prediction performance of the model. MSE represents the average of the squared error between the predicted and actual values. MAE measures the average absolute error between the predicted and actual values. MAE is typically employed to evaluate the predictive accuracy of regression models, reflecting the average size of the absolute value of errors in the predictions. In comparison with other evaluation index, such as MSE, MAE is more robust to outliers.

#### Prediction models

In this study, four machine learning algorithms (XGBoost, Random forest, SVM, and BP neural network) are employed to construct the prediction model. The principles are as follows:

XGBoost is a gradient boosting algorithm based on decision trees [15]. The core concept of gradient boosting involves training a series of weak learners (such as decision trees) and combining their predicted results through weighted majority voting. XGBoost can also automatically adjust the weak learner parameters using internal cross-validation. In this study, grid search under 5-fold cross-validation is utilized to determine the value of the hyperparameters. In order to facilitate the comparison of the prediction results of various models, after multiple experiments, the least squares loss was selected as the objective function, the XGBoost model in this paper was established by using the number of base learners as 18–19, the seed value of random number generator as 20, the learning rate as 0.19, and the proportion of samples taken in random sampling as 0.92–0.93.

The random forest algorithm is an ensemble learning method based on decision trees, essentially a bagging ensemble algorithm [16]. For classification problems, each tree outputs a classification result, and the random forest classification result is the mode of the classification results from each tree. For regression problems, each tree outputs a regression result, and the random forest regression result is the average of the regression results of all trees. In this study, after multiple experiments, a random forest model was established using 92–93 base learners, a seed value of 20 for the random number generator, a maximum tree depth of 16, and a minimum sample leaf node size of 6.

Support Vector Machine (SVM) is a supervised learning algorithm applicable for classification or regression tasks. The objective of SVM is to identify the hyperplane that maximally separates positive and negative classes in high-dimensional space [17]. There are three key parameters of SVM model: kernel function, penalty coefficient of soft interval and gamma value. By using grid search method, this paper established SVM prediction model by taking Gaussian kernel function, penalty coefficient of soft interval as 1 and gamma parameter as ’auto’.


$$K(x,y)=x^{T}y=x\cdot y$$ (4)

$$K(x,y)={\left(\gamma (x\cdot y)+r\right)}^{d}$$ (5)

K(x,y)=tanh(γ(x⋅y)+r) (6)

$$K(x,y)=e^{-\gamma {\left\|x-y\right\|}^{2}},\gamma >0$$ (7)

**Fig 5:**
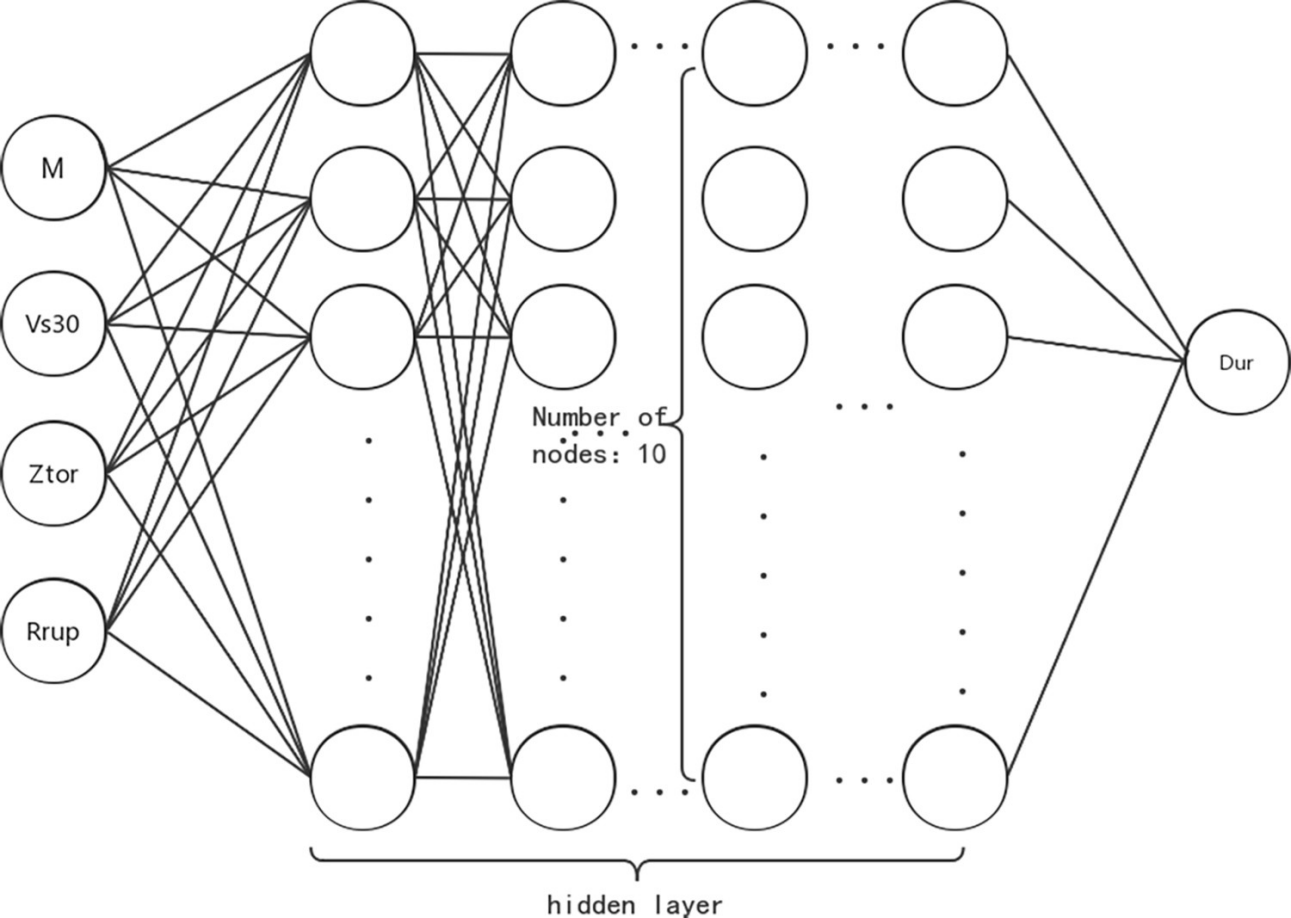
BP neural network structure diagram.

The Back Propagation (BP) neural network algorithm is an artificial neural network learning algorithm based on the error back propagation algorithm, consisting primarily of three steps: forward propagation, back propagation, and weight updating [18]. In this study, the BP neural network model includes an input layer, an output layer, and four hidden layers, with 10 nodes in each hidden layer (Fig 5).

To eliminate the influence of different parameter scales on SVM and BP neural networks, seismic data must undergo standardization for dimensionless processing. The formula is as follows:

$$x^{*}=\frac{x-\mu }{\sigma }$$ (8)

After dimensionless processing, the data are divided into training and test sets, with 85% of the data designated as the training set and 15% as the test set.

**Fig 6:**
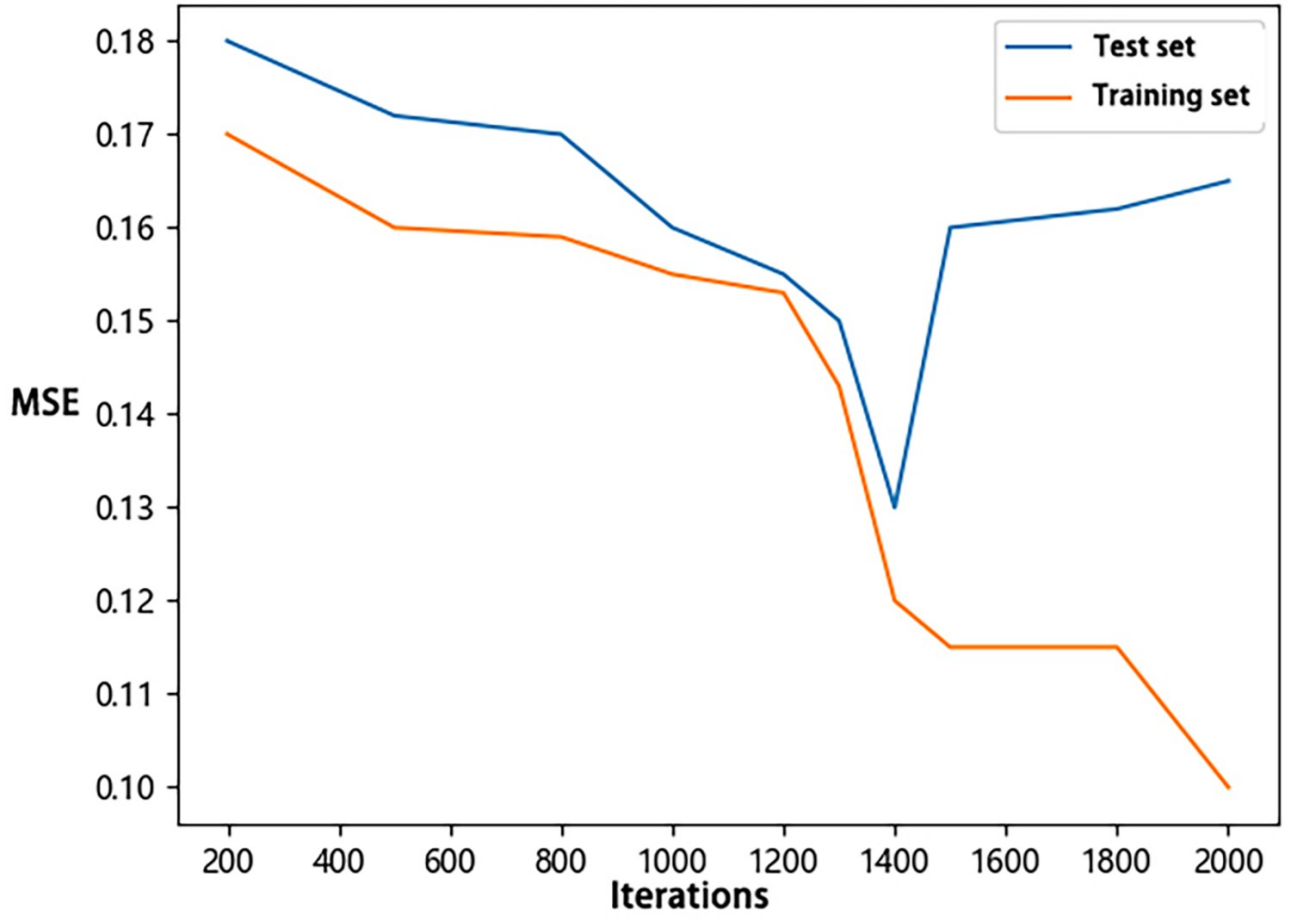
MSE variation with iterations number for *D*_*5-95*_.

**Fig 7:**
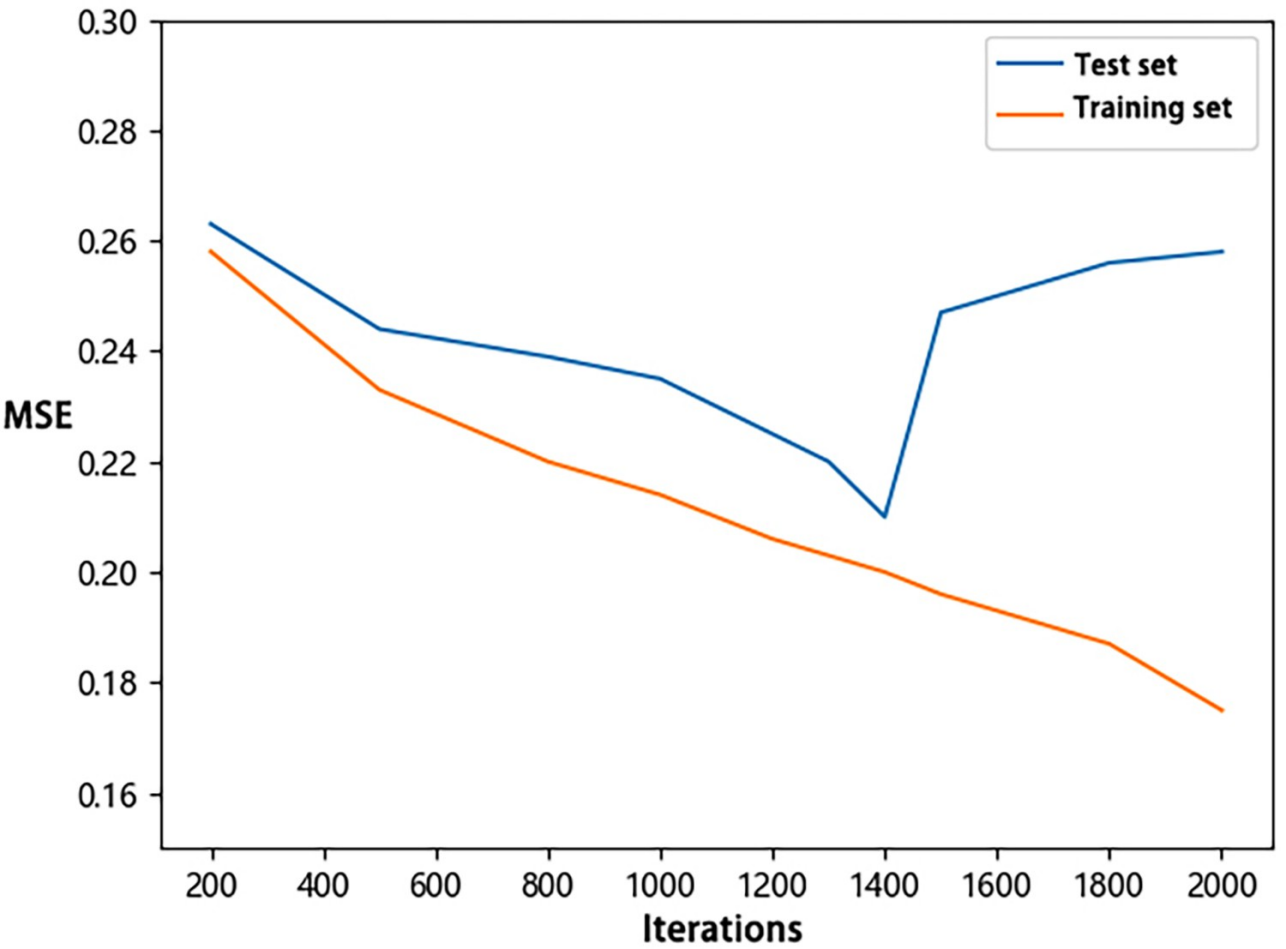
MSE variation with iterations number for *D*_*5-75*_.

For the construction of the *D*_5-95_ and *D*_5-75_ BP neural networks, a learning rate of 0.01 was employed. The networks were trained with various numbers of iterations (200, 500, 800, 1000, 1200, 1300, 1400, 1500, 1600, 1800, and 2000) for 20 runs to select the optimal model. Optimal models at different iteration numbers were selected, and the MSE of the training and test sets were presented in line charts (Figs 6 and 7). It can be observed that for both *D*_5-95_ and *D*_5-75_, the MSE of both the test and training sets decreased before 1400 iterations. After 1400 iterations, the training set MSE continued to decrease, while the test set MSE increased, indicating the model began to overfit. Consequently, 1400 iterations were chosen as the optimal number of iterations for both *D*_5-95_ and *D*_5-75_ neural networks.

#### Combination analysis of characteristic parameters

**Fig 8:**
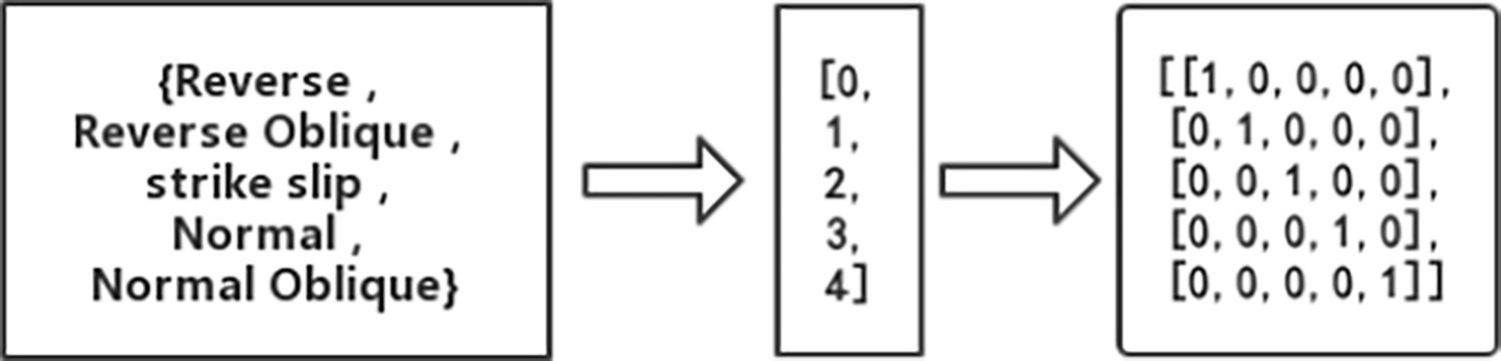
Unique thermal code for epicenter mechanism parameters.

The most commonly characteristic parameters of significant duration are moment magnitude(*M*_w_), fault distance (*R*_rup_) and average shear wave velocity(*V*_S30_). In this study, the fault-to-surface depth parameter (*Z*_tor_) and epicenter mechanism parameter (Mec) were introduced for characteristic parameter optimization analysis of the prediction model. The epicenter mechanism encompasses Reverse, Reverse Oblique, Strike Slip, Normal, and Normal Oblique. In this study, the epicenter mechanism parameters are created as dummy variables by means of unique thermal code, and XGBoost is used for parameter combination analysis, as illustrated in Fig 8.

Four parameter combinations were formed: *M*_w_&*R*_rup_&*V*_S30_, *M*_w_&*R*_rup_&*V*_S30_&Mec, *M*_w_&*R*_rup_&*V*_S30_0&*Z*_tor_, and *M*_w_&*R*_rup_&*V*_S30_&*Z*_tor_&Mec. The XGBoost algorithm was selected for characteristic parameter optimization analysis. MSE and MAE were utilized as evaluation indicators to select the best-performing parameter combination. Table 1 displays the comparison results on the test set under 5-fold cross-validation. The following observations can be made:

After incorporating the epicenter mechanism (Mec) into the model, the MSE and MAE decreased slightly. For the prediction of *D*_5-75_, the MSE value and MAE value in test set decreased by 1.8% and 1.1% respectively. For the prediction of *D*_5-95_, the MSE value and MAE value in test set decreased by 2.5% and 0.72% respectively.

After introducing *Z*_tor_ into the XGBoost model, the MSE value and MAE value are both decreased significantly. For the prediction of *D*_5-75_, the MSE value and MAE value in test set decreased by 7.2% and 3.6% respectively. For the prediction of *D*_5-95_, the MSE value and MAE value in test set decreased by 6.9%, and 2.5% respectively.

Comparing the prediction results of the four-parameter combination(*M*_w_&*R*_rup_&*V*_S30_&*Z*_tor_) and the five-parameter combination (*M*_w_&*R*_rup_&*V*_S30_&*Z*_tor_&Mec), the MSE and MAE values of *D*_5-75_ and *D*_5-95_ are the same on the test set, which has not improved the predictive accuracy of significant duration.

As stated in the review, the four-parameter combination (*M*_w_&*R*_rup_&*V*_S30_&*Z*_tor_) was chosen as the foundation for subsequent research in this study.

**Table 1:** Comparison of different parameter combinations of significant duration (test set).

Parameter combination (Using XGBoost)	*D*_*5-75*_		*D*_*5-95*_	
	MSE	MAE	MSE	MAE
*M*_*w*_ *&R*_*rup*_ *&V*_*s30*_	0.221	0.360	0.13	0.276
*M*_*w*_*&R*_*rup*_*&V*_*s30*_*&*Mec	0.217	0.356	0.127	0.274
*M*_*w*_ *&R*_*rup*_ *&V*_*s30*_ *&Z*_*tor*_	0.205	0.347	0.121	0.269
*M*_*w*_*&R*_*rup*_*&V*_*s30*_*&Z*_*tor*_*&*Mec	0.205	0.347	0.121	0.269

#### Result and analysis

The generalization abilities of the four machine learning models were assessed using MSE and *R*^2^. Tables 2 and 3 present the evaluation index values of *D*_5-95_ and *D*_5-75_ for the four machine learning algorithms on the training and testing sets. In Table 2, the random forest model exhibited a lower MSE than the other three models, with reductions of 4.3%, 29.9%, and 39.4% respectively on the training set, with reductions of 3.3%, 10%, and 22% respectively on the testing set. In Table 3, the random forest model displayed a lower MSE than the other three prediction models on both the training and test sets, the MSE value in training set decreased by 6.4%, 34%, and 45% respectively. the MSE value in test set decreased by 2.4%, 4.8%, and 17.7% respectively. The *R*^2^ of the random forest and XGBoost models were similar, and the random forest model demonstrated slightly better fitting performance than the XGBoost model; however, both were significantly better than the BP neural network and SVM models.

**Table 2:** Data comparison of different models for *D*_5-95_.

Models	*R* ^*2*^_training_	*R* ^*2*^_test_	MSE_training_	MSE_test_
Random forest	0.81	0.73	0.089	0.117
XGBoost	0.80	0.72	0.093	0.121
BP Neural network	0.72	0.69	0.127	0.130
SVM	0.65	0.64	0.147	0.15

**Table 3:** Data comparison of different models for *D*_*5-75*_.

Models	*R* ^2^_training_	*R* ^2^_test_	MSE_training_	MSE_test_
Random forest	0.81	0.71	0.132	0.2
XGBoost	0.79	0.70	0.141	0.205
BP Neural network	0.70	0.68	0.2	0.21
SVM	0.66	0.65	0.24	0.243

In summary, the random forest model demonstrated the best prediction performance, followed by XGBoost, while SVM performed the worst.

To more intuitively assess the fitting performance of the models, model residuals were employed to compare the actual and predicted values of significant duration. The model residual was defined as the difference between the natural logarithms of the measured and predicted seismic duration, with the formula as follows:

$$\eta (Dur)=\ln(Dur_{true})-\ln(Dur_{pred})$$ (9)

Where: ln(*Dur*_*true*)_ is the measured value of ground motion duration, and ln(*Dur*_*pred*)_ is the predicted value of ground motion duration.

**Fig 9:**
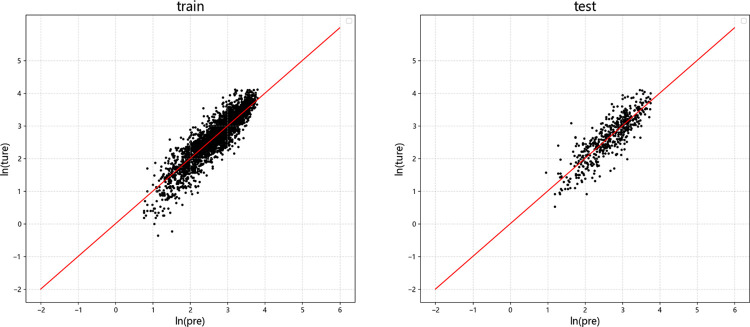
Residual graph of *D*_5-95_ predicted by random forest. (a) Training set (b)Test set.

**Fig 10:**
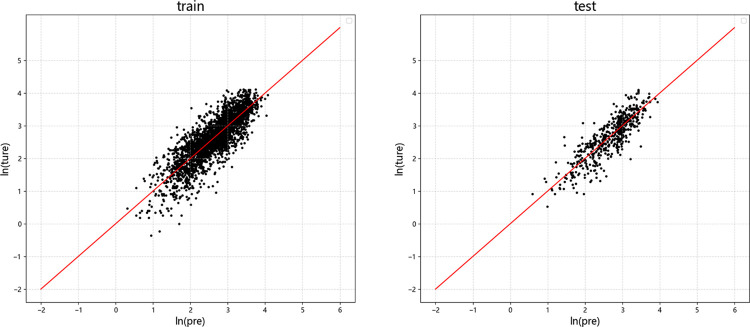
Residual graph of *D*_5-95_ predicted by BP Neural Network. (a) Training set (b)Test set.

**Fig 11:**
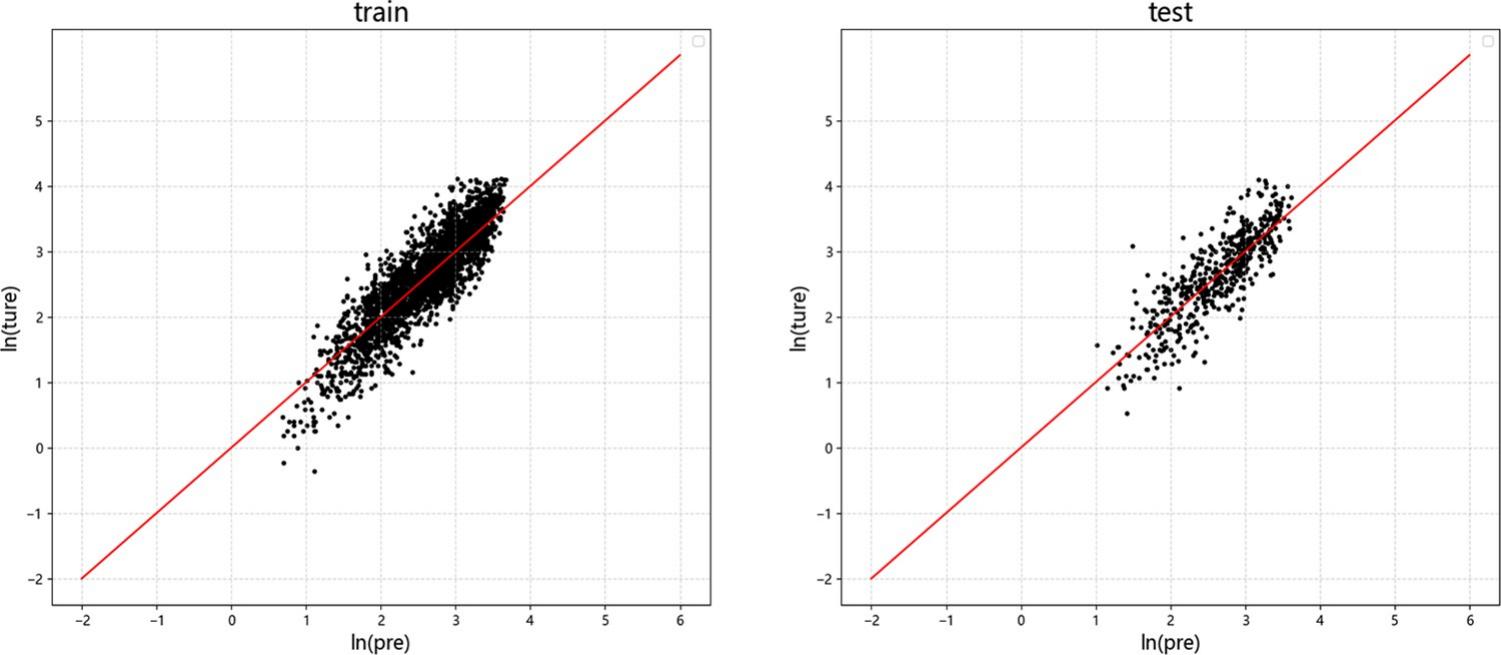
Residual graph of *D*_5-95_ predicted by XGBoost. (a) Training set (b)Test set.

**Fig 12:**
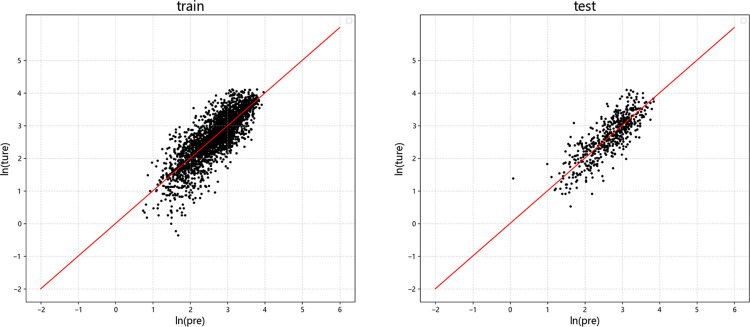
Residual graph of *D*_5-95_ predicted by SVM. (a) Training set (b)Test set.

Figs 9–12 display the residual plots of the significant duration for the four machine learning models using *D*_5-95_ as an example. The following observations can be made:

The random forest, BP neural network, and XGBoost models exhibited good fitting performance for predicting *D*_5-95_. The SVM model has poor fit.

When predicting *D*_5-95_, the random forest, BP neural network, and XGBoost models show the tend of underestimate large values and overestimate small values.

In the test set, there is no significant difference between the results of *D*_5-95_ predicted by random forest, neural network and XGBoost model.

### Proposed model fusion

#### Fusion model modelin

To further enhance the generalization performance of the models and reduce the generalization error of individual models, through the verification of multiple model combinations, the prediction accuracy of three models (random forest, XGBoost, BP neural network) for model fusion is the highest.javascript:void(0);

At present, the main methods for model fusion include stacking method and weighted average method.

Stacking is a commonly used method in model fusion, which entails combining multiple models, using their outputs as inputs for a new model, and employing the new model to generate the final prediction results [19].

**Fig 13:**
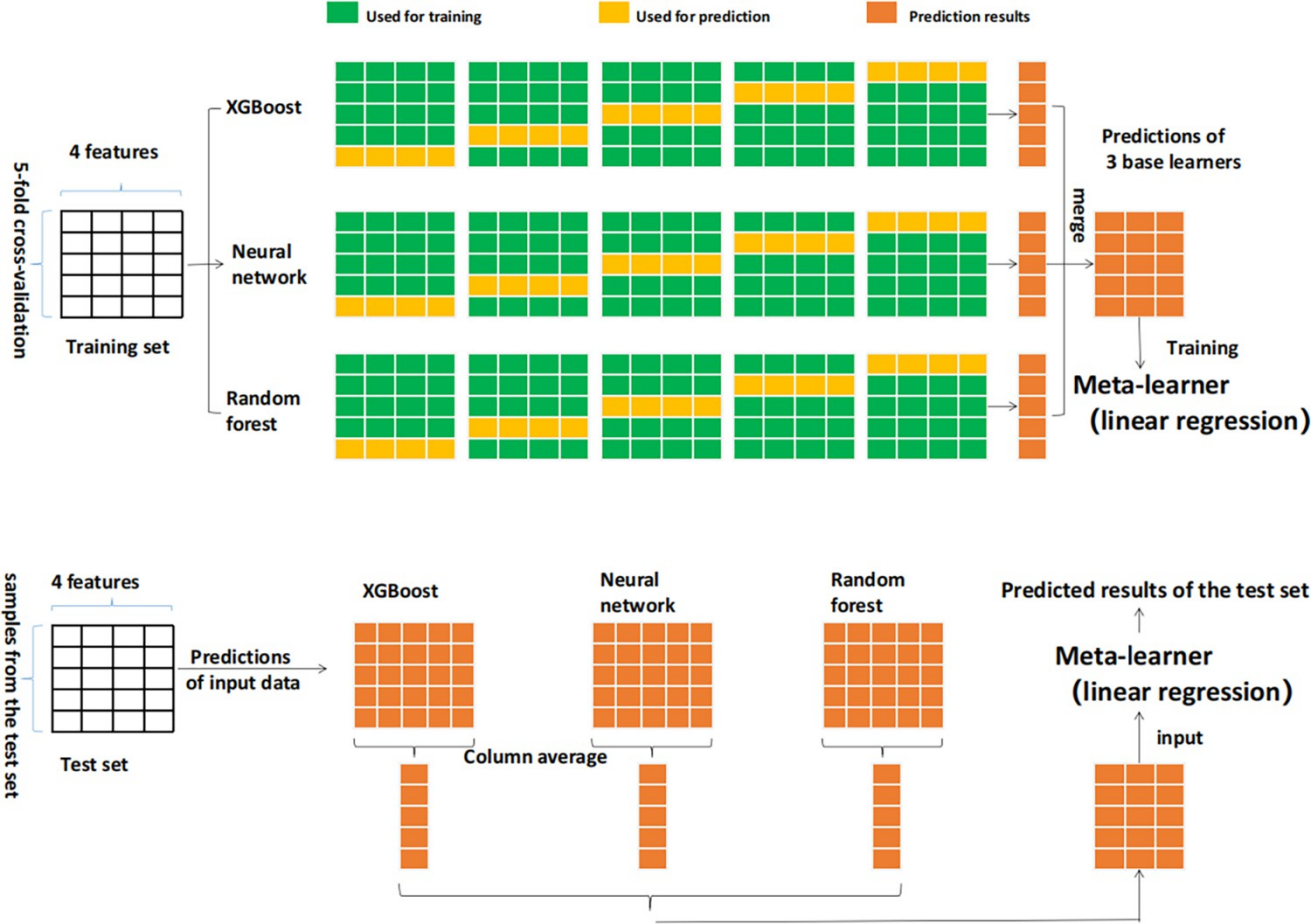
Schematic diagram of stacking method.

The framework and process of the Stacking fusion model (Fig 13) are as follows:

Firstly, Utilize Random Forest, XGBoost, and BP neural network as the first-layer base learners.

Secondly, Employ a linear regression model as the second-layer meta-learner.

At last, Five-fold cross-validation was adopted for two-layers learners, and MSE was used as the tuning balance index.

**Fig 14:**
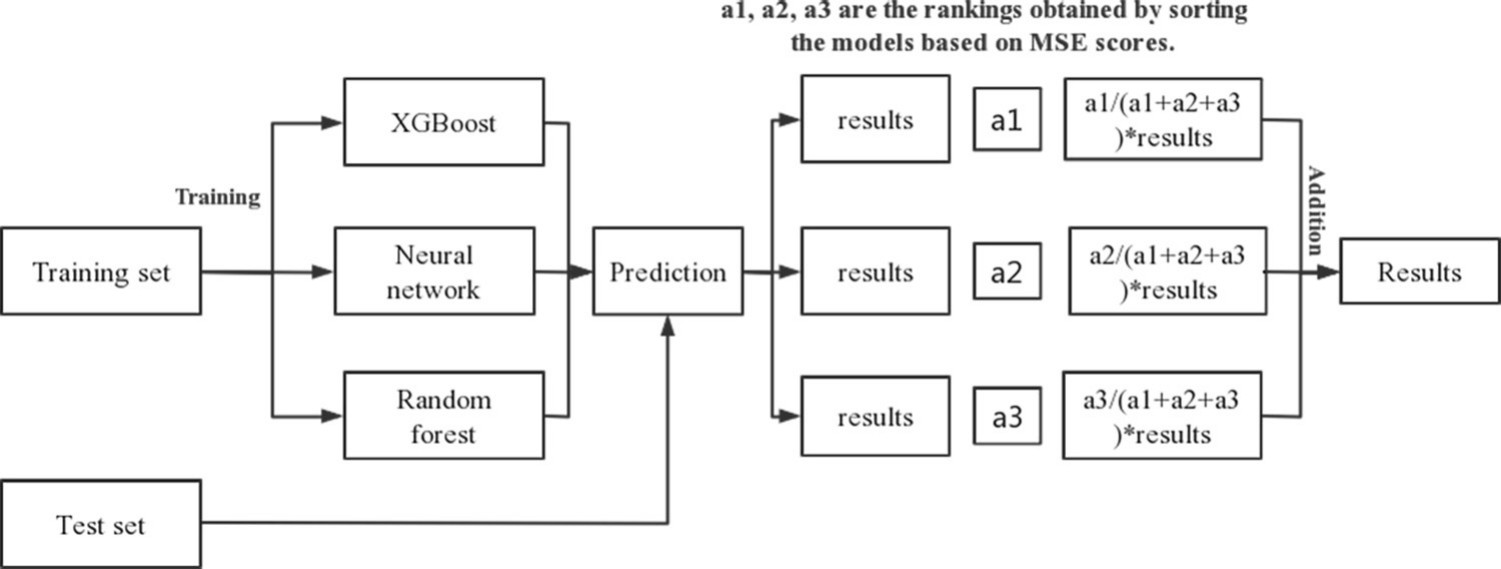
Principle of weighted average method model fusion.

Weighted averaging correlates the weights of the models with their performance, assigning higher weights to models with better performance and contributing more significantly to the overall prediction. The process of the Weighted Averaging fusion model (Fig 14) involves ranking MSE scores of the three individual models (Random Forest, XGBoost, BP neural network) in ascending order. Smaller MSE scores receive higher rankings, and weights are allocated based on the rankings. Subsequently, the predicted results and the assigned weight coefficients of the individual models are used to obtain the prediction results of the fusion model.

#### Prediction results of fusion model

Tables 4 and 5 present a comparative evaluation of significant duration prediction results between the fusion model and individual models. The MSE of the fusion model on the test set is smaller than that of the single models, indicating that the fusion model exhibits better fitting performance than the individual models. Moreover, the fusion model employing the weighted averaging method achieves the best prediction performance. The *R*^2^ on the test set increases and the MSE decreases. The evaluation index *R*^2^ of the fusion model (weighted averaging method) on the test set is 0.77, closely approaching the training set’s 0.81, which suggests that the model has reached a point of low generalization error.

**Table 4:** Comparison of *D*_5-95_ prediction results of fusion model and single model.

Prediction model	*R* ^*2*^_training_	*R* ^*2*^_test_	MSE_training_	MSE_test_
Random forest	0.81	0.73	0.089	0.117
XGBoost	0.79	0.72	0.093	0.121
BP neural network	0.72	0.69	0.12	0.13
Fusion model (Stacking method)	0.81	0.72	0.092	0.115
Fusion model (weighted average method)	0.81	0.77	0.095	0.107

**Table 5:** Comparison of *D*_5-75_ prediction results of fusion model and single model.

Models	*R* ^*2*^_training_	*R* ^*2*^_test_	MSE_training_	MSE_test_
Random forest	0.81	0.71	0.132	0.2
XGBoost	0.79	0.70	0.141	0.205
BP neural network	0.70	0.68	0.2	0.21
Fusion model (Stacking method)	0.81	0.72	0.12	0.186
Fusion model (weighted average method)	0.81	0.75	0.128	0.167

**Fig 15:**
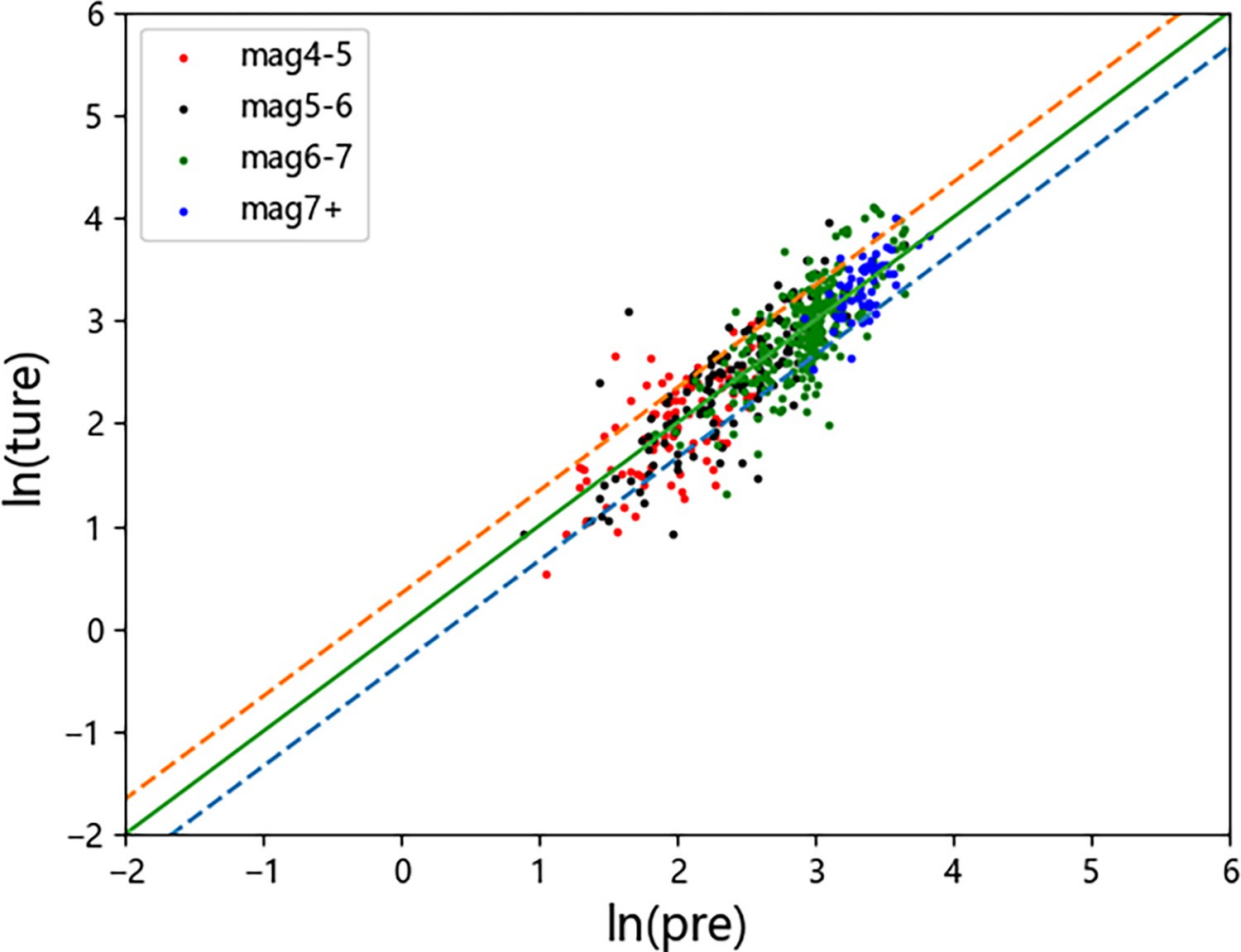
*D*_*5-95*_ residual plot (test set).

**Fig 16:**
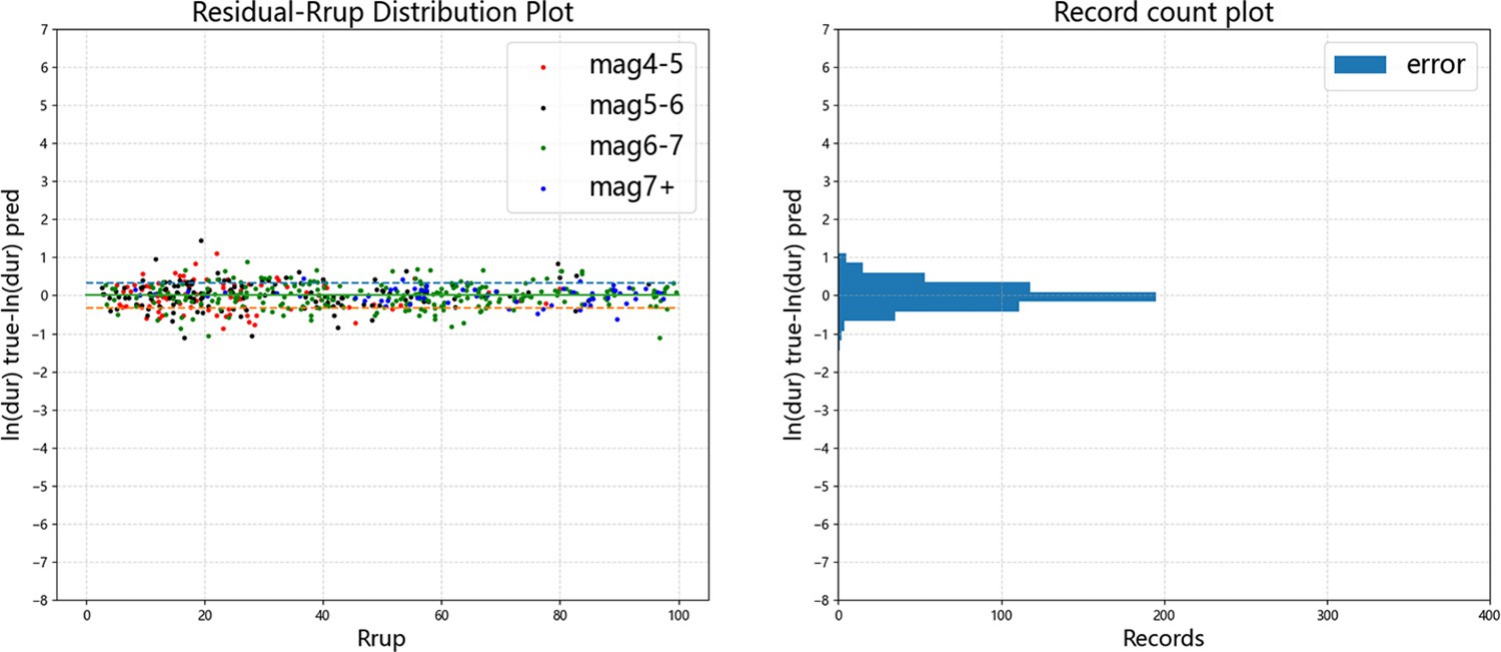
Error distribution of *D*_*5-95*_ error versus fault distance (test set).

**Fig 17:**
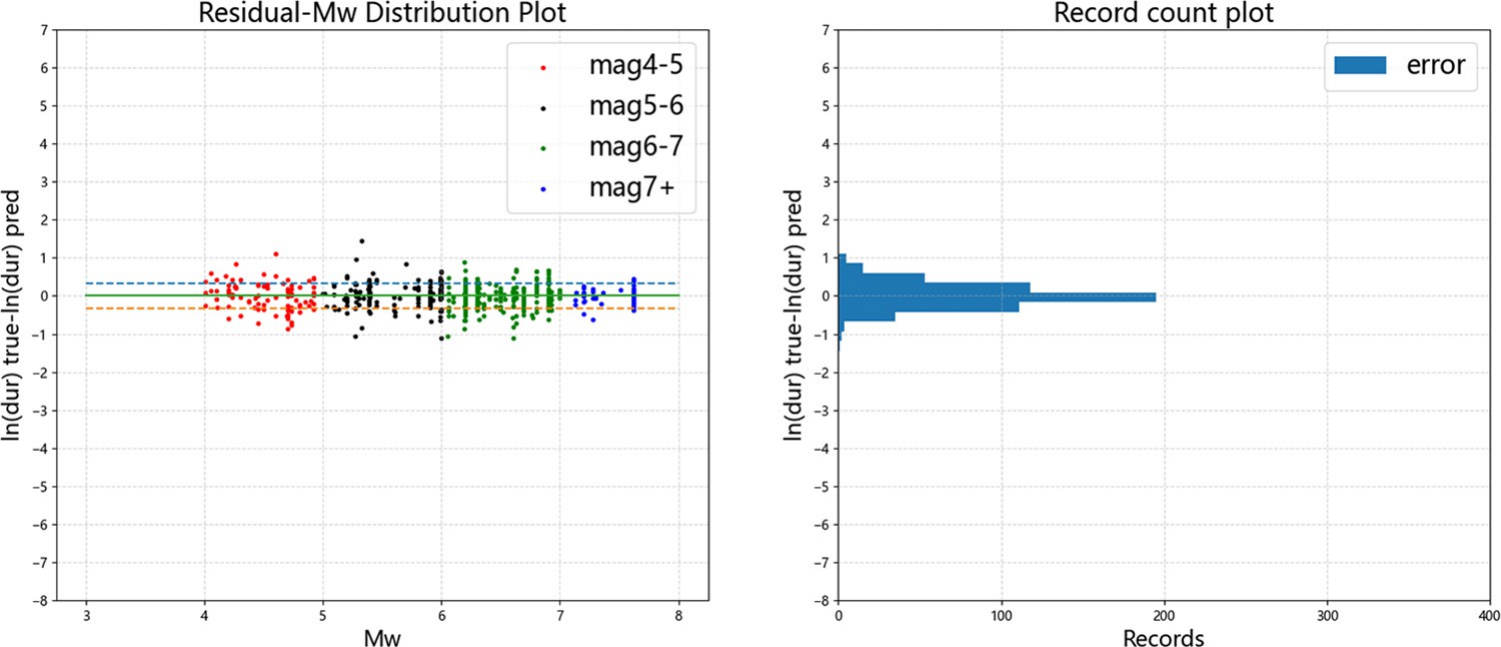
Error distribution of *D*_*5-95*_ error versus moment magnitude (test set).

**Fig 18:**
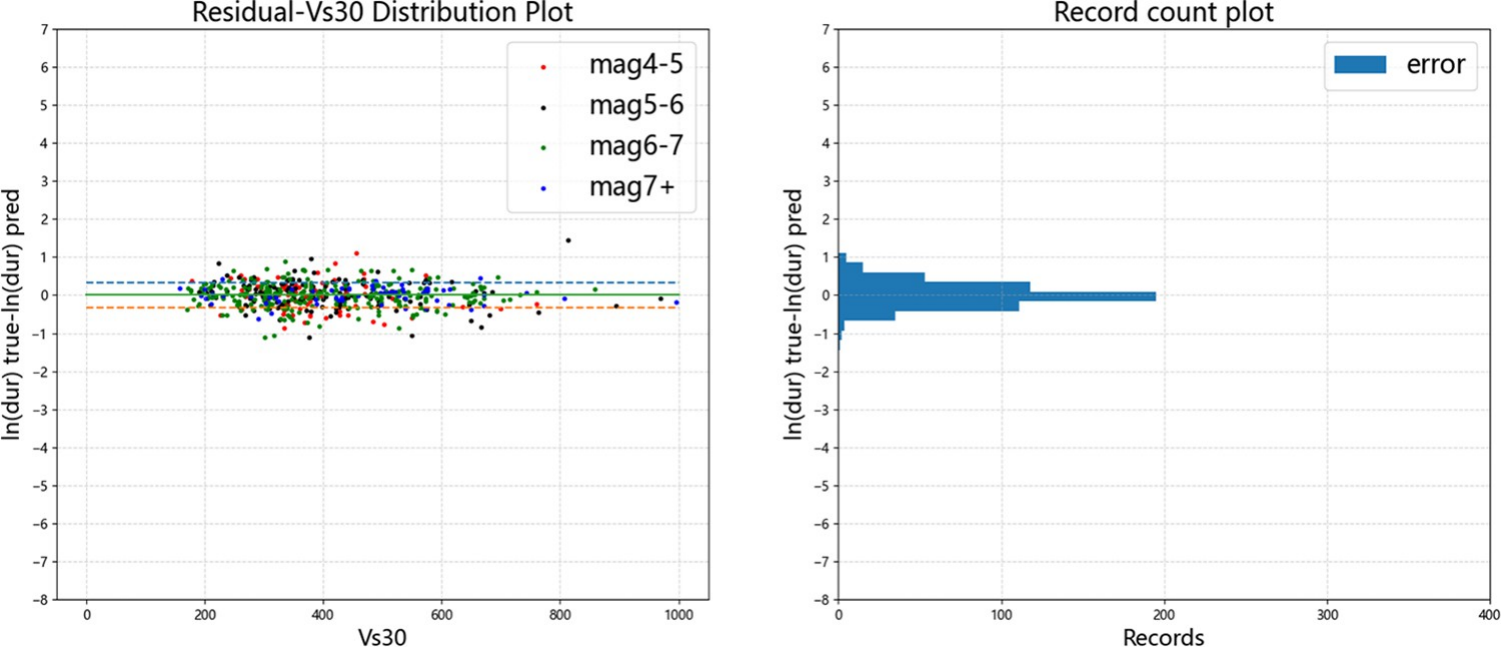
Error distribution of *D*_*5-95*_ error versus *V*_*S30*_ (test set).

**Fig 19:**
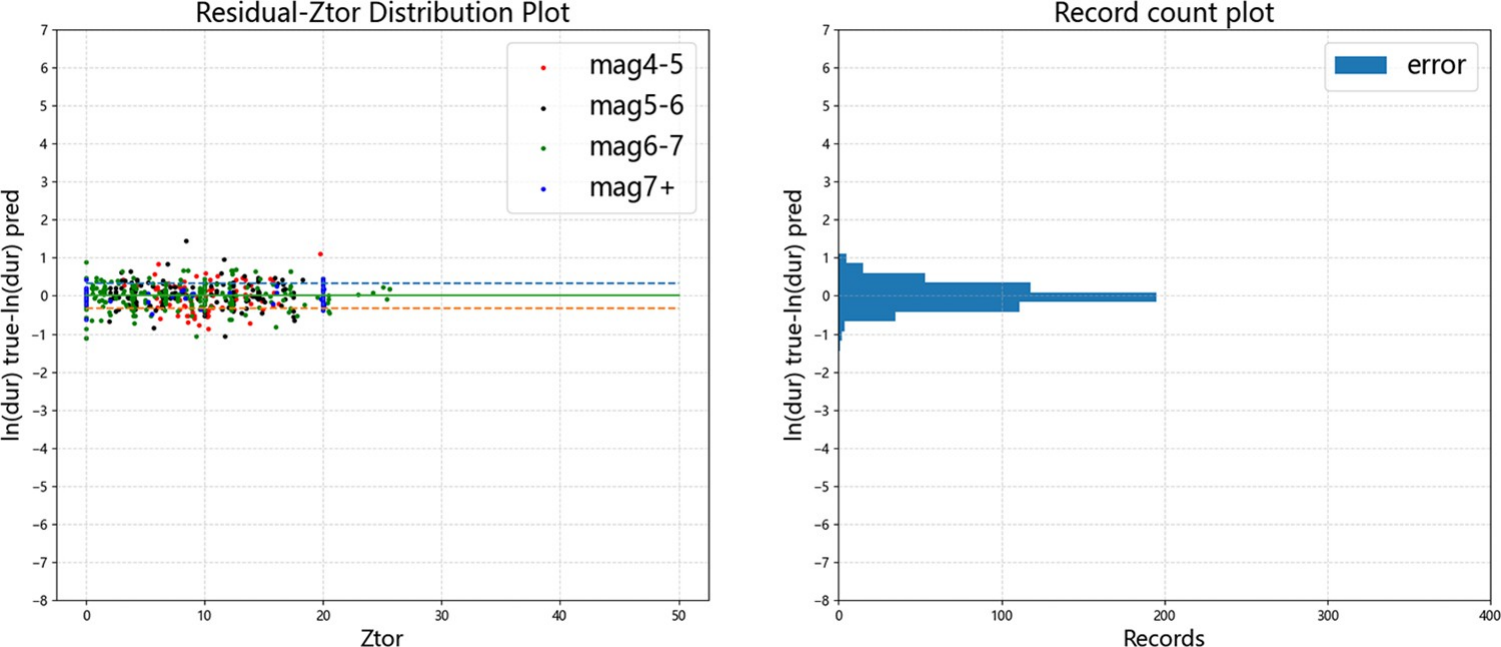
Error distribution of *D*_*5-95*_ error versus *Z*_*tor*_ (test set).

Figs 15–19 display the trends of the significant duration prediction residuals of the fusion model on the test set. *D*_5-75_ and *D*_5-95_ exhibit similar residual patterns in the test set. So analysis results are provided using *D*_5-95_ duration.

The significant duration increases with moment magnitude, and the residuals are approximately normally distributed. They are uniformly distributed on both sides of the fault distance, moment magnitude *V*_s30_, and *Z*_tor_ zero line, indicating that the fusion model has a high degree of fit. The dashed line represents one standard deviation between predicted value and actual value of 0.326 for ln(*D*_5-95_), and the data mostly falls within one standard deviation. The average residual value is 0.00054, which is close to the theoretical value of 0.

#### Comparison with existing studies

To verify the effectiveness of the proposed fusion model, we compared its significant duration predictions with those of Stafford [6] and Wang [20], both of which were based on the same data source (NGA-WEST2). Table 6 provides numerical comparisons of model evaluation indexs, including MSE, MAE, and error rate.

**Table 6:** Compared with the existing prediction equation.

Models	*D*_5-95_ duration			*D*_5-75_ duration		
	MSE	MAE	Error rate	MSE	MAE	Error rate
Stafford(2009)	0.179	0.333	12.50%	0.283	0.407	21.80%
Wang(2021)	0.177	0.329	12.40%	0.306	0.417	22.40%
Proposed fusion model (weighted average method)	0.107	0.249	9.10%	0.168	0.313	16.20%

As demonstrated in Table 6, for the prediction of significant duration *D*_5-95_, compared to the models of Stafford [6] and Wang [20], the proposed fusion model (weighted averaging method) reduced the MSE value by 40.2% and 39.5% respectively, and decreased the MAE value by 25.2% and 24.3% respectively, while enhancing the overall accuracy by 3.4% and 3.3% respectively. For the prediction of significant duration *D*_5-75,_ compared to the models of Stafford and Wang, the proposed fusion model (weighted averaging method) reduced the MSE by 40.6% and 45% respectively, and decreased the MAE by 23.1% and 24.9% respectively, while improving the overall accuracy by 5.6% and 6.2%. Therefore, compared to existed fitting methods, the proposed significant duration fusion model (weighted averaging method) improves prediction accuracy and significantly reduces prediction errors.

## Conclusions

1. By incorporating *Z*_tor_ and epicenter mechanism parameters, and utilizing XGBoost for modeling, an optimal characteristic parameter combination (*M*_w_&*R*_rup_&*V*_s30_&*Z*_tor_) was identified for predicting significant duration based on MSE and MAE evaluation index.
2. Prediction models for the significant duration of seismic motion were developed using random forest, XGBoost, BP neural network, and SVM. Evaluation of the models using *R*^2^, MSE, MAE, and residual analysis of significant duration (*D*_5-75_ and *D*_5-95_) demonstrated that random forest and XGBoost provided superior fitting effects for both the training and testing sets, whereas SVM exhibited the poorest prediction performance.
3. Model fusion techniques, such as stacking and weighted averaging, were employed to combine the prediction models. By contrasting the predictive outcomes of individual models and fusion models, the weighted averaging fusion model displayed a more substantial improvement, with its *R*^2^ value approaching that of the training set, resulting in a reduced generalization error.
4. To assess the effectiveness of the fusion model, it was compared with existing models. The proposed weighted averaging fusion model, incorporating the four characteristic parameters of *Z*_tor_, demonstrated smaller prediction errors and enhanced prediction accuracy for significant duration of *D*_5-75_ and *D*_5-95_.

It has also been noted that the factors affecting the prediction of significant duration of ground motion may not only be part of the characteristic parameters considered in this paper, such as the directional effect of fault rupture, deep velocity structure, etc., and the effective quantitative measures of these factors need to be further studied in the future work. In addition, the generalization ability and applicability of the fusion prediction model proposed in paper may also be affected by the division method of training set and test set in the data set, and its influence mechanism and law need to be further analyzed.

## Supporting information

S1 File (ZIP)
